# Les cellulites cervico-faciales graves, facteurs et critères de gravité

**DOI:** 10.11604/pamj.2014.18.57.3702

**Published:** 2014-05-15

**Authors:** Mohammed Lakouichmi, Khalid Tourabi, Bader-eddine Abir, Said Zouhair, Saad Lahmiti, Nadia Mansouri Hattab

**Affiliations:** 1 Service de Chirurgie Maxillo Faciale Hôpital Militaire Avicenne, Marrakech, Maroc; 2 Service de Bactériologie Hôpital Militaire Avicenne, Marrakech, Maroc

**Keywords:** Cellulites, graves, critères de gravités, facteurs favorisants, cellulitis, severe, severity criteria, predisposing factors

## Abstract

La cellulite cervico-faciale grave est une infection polymicrobienne extensive et redoutable du tissu cellulo-adipeux de la face et du cou. L'objectif de cette étude est d'analyser certains facteurs favorisants et d’évaluer les critères de gravité en fonction des formes anatomo-cliniques. Il s'agit d'une étude rétrospective réalisée, entre janvier 2007 et décembre 2012, au service de chirurgie maxillo faciale de l'hôpital militaire Avicenne de Marrakech. Sur 147 cas de cellulites cervico-faciales pris en charge au niveau du service, 13 dossiers de cellulites graves ont été retenus. Neuf hommes (69%) et quatre femmes (31%) ont fait l'objet de cette étude, avec un âge moyen de 35 ans. Tous les patients ont été adressés pour prise en charge secondaire après avoir pris des anti-inflammatoires (AI). Sept cas (54%) étaient immunocompétents. La cause dentaire était soulevée chez neufs cas (69%). Cinq cas (38%) ont présenté une forme pseudo phlegmoneuse avec des signes compressifs des voies aéro-digestives. L'extension médiastinale a été observée chez quatre patients (31%). La forme nécrosante extensive a été retrouvée dans trois cas (23%). L’étude bactériologique, réalisée chez tous les patients, avait mis en évidence une flore microbienne mixte et polymorphe. Les cellulites cervico-faciales graves posent un réel problème de prise en charge thérapeutique. L'analyse des facteurs favorisants et l’évaluation des critères de gravité dans cette série ont permis de limiter une évolution défavorable.

## Introduction

Les cellulites cervico-faciales graves sont des affections brutales, redoutables et posent un sérieux problème de prise en charge thérapeutique. Peu fréquentes, ces complications peuvent compromettre le pronostic vital et constituent un véritable problème de santé public [1, 2]. Le plus souvent ces cellulites surviennent, soit sur un terrain fragile, soit à l'issue d'une prise en charge initiale inappropriée. Le but de ce travail est d'analyser certains facteurs favorisants et d’évaluer les critères de gravité.

## Méthodes

Il s'agissait d'une étude rétrospective réalisée, entre janvier 2007 et décembre 2012, au service de chirurgie maxillo faciale de l'hôpital militaire Avicenne de Marrakech. Sur 147 cas de cellulites cervico-faciales pris en charge au niveau du service 13 dossiers de cellulites graves ont été retenus. Pour chaque dossier ont été relevés: l’âge, le sexe, le terrain, le siège, la prise d'anti-inflammatoires (AI), la porte d'entrée, les signes cliniques de gravité, les germes en causes et l’évolution.

## Résultats

**Figure 1:**
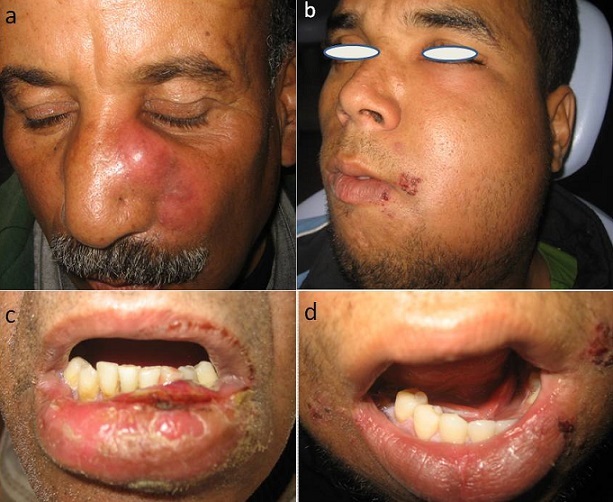
a) cellulite naso-génienne avec risque de thrombo-phlébite; b) cellulite génienne gauche diffuse; c) cellulite à point de départ muqueux; d) pseudo-phlegmon du plancher buccal

L’étude est réalisée sur 13 cas, neufs hommes (69%) et quatre femmes (31%). Tous les patients ont été adressés pour prise en charge secondaire après échec du traitement initial et évolution défavorable. L’âge moyen était de 35 ans avec des extrêmes de 21 ans et 57 ans. A l'admission tous les patients avaient déjà pris des AI et un ou plusieurs antibiotiques. Sept cas (54%) ne présentaient aucune tare, trois patients (23%) étaient diabétiques et trois (24%) immunodéprimés. La cause dentaire était soulevée chez neufs cas (69%), le traumatisme par effraction muqueuse ou cutanée chez quatre cas (31%). Les signes cliniques: trismus serré, troubles compressives des voies aéro-digestives supérieures (dysphagie, dyspnée modérée), crépitations neigeuses sous-cutanées, douleur thoracique en plus des signes habituels de la cellulite cervico-faciale (Figure 1), ont été rencontrés chez les patients différemment selon la localisation (Tableau 1). Quatre patients ont présenté une médiastinite (31%), trois (23%) une nécrose cutanée, une ostéite du ramus, un cas de septicémie et une patiente a présenté des fistules multiples du scalp. La température variait entre 38° et 39,5°.

**Tableau 1:** Signes cliniques de gravité selon la localisation anatomique des différents cas étudiés

Nombre cas	Ages (année)	sexe	terrain	localisation	Prise IA	Signes cliniques	cause	évolution
1	32	F	0	Parotido-masséterine	+	Trismus serré	Dent	Ostéite ramus
2	40	M	0	Plancher buccal	+	Trismus, dysphagie	Dent	Favorable
3	25	M	0	Génienne	+	Crépitations	Dent	Nécrose cutanée
4	46	F	diabète	Parotido-masseterine	+	Trismus, nécrose	Dent	Fistules scalp
5	28	F	0	Cervico faciale	+	Douleur thoracique	Dent	Médiaastinite
6	37	M	diabète	Cervico thoracique	+	Crépitations, douleur	Dent	Médiaastinite
7	21	M	0	Génienne	+	Crépitations	Dent	Nécrose cutanée
8	33	M	0	Plancher buccal	+	Trismus, dysphagie	Plaie langue	Favorable
9	36	M	diabète	Cervico thoracique	+	Crépitations, douleur	dent	Médiaastinite
10	57	F	aplasie médullaire	Plancher buccal	+	Dyspnée, dysphagie	Plaie labiale	Septicemie
11	32	M	toxicomane	Pan faciale	+	Crépitations	Plaie	Sequelles innesthétiques
12	41	M	IRC	Cervico thoracique	+	Douleur thoracique	Dent	Médiastinite
13	31	M	0	Submentale	+	crépitations	Plaie labiale	Nécrose cutanée

Abréviations: F,féminin; M, masculain; IRC, insufusance rénale chronique; 0, immunocompétent; AI, anti-inflammatoire; +, utilisation d'anti-inflammatoires.

**Figure 2:**
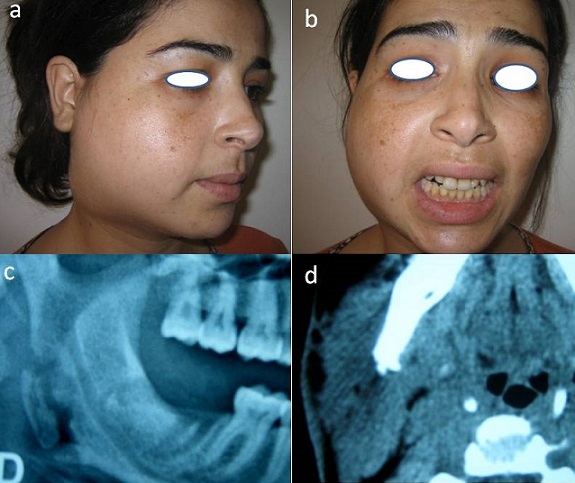
a) phlegmon de la loge massétro-parotidienne droite; b) trismus serré; c) ostéite du ramus avec séquestre osseux sur l'orthopantomogramme; d) aspect scanographique (en coupe axiale) avec infiltration cellulitique de la loge masséterique

**Figure 3:**
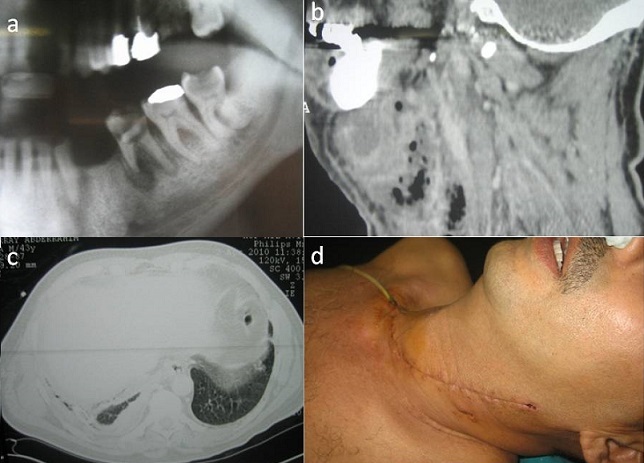
a) granulome inflammatoire de la 36 (dent causale) sur l'orthopantomogramme; b: infiltration cellulitique du tissu cellulo-graisseux cervical sur la tomodensitométrie (TDM) en reconstruction sagittale; c) comblement médiastinale (TDM en coupe axiale); d) drainage médiastinale

L'hyperleucocytose était modérée entre 12000 et 13000 éléments /mm3 sauf dans un cas d'aplasie médullaire. L’étude bactériologique réalisée chez tous les cas (prélèvements parfois écho-guidés) avait montré une flore bactérienne mixte, polymorphe aérobie et anaérobie avec le plus souvent l'association de plusieurs germes dont certains étaient multi résistants à l'antibiogramme (Tableau 2). L'orthpantomogramme et la tomodensitométrie (maxillo faciale, cervico médiastinale et parfois thoracique) ont été réalisés chez l'ensemble des malades pour rechercher la cause et apprécier le degré d'extension. Ils avaient monté une ostéite du ramus avec séquestre (Figure 2) et trois médiastinite (Figure 3). Le traitement était médicochirurgical. L'antibiothérapie initiale associait amoxicilline- acide clavulanique gentamycine puis elle était adaptée en fonction des germes retrouvés: six cas (46%) avaient eu amoxicilline-acide clavulanique-métronidazol-gentamycine, quatre cas (31%) céfotaxime-métronidazol-ciprofloxacine, et trois cas (23%) teicoplanine-amoxicilline-acide clavulanique. La durée moyenne du traitement antibiotique était de trois semaines sauf dans un cas où elle a été prolongée à huit semaines (ostéite actinomycosique). L'intubation était orotrachéale chez dix patients (77%) et dans trois cas (23%) à l'aide d'un naso-fibroscope. Le traitement chirurgical (traitement de la cause + drainage et/ou débridement) était réalisé chez tous les patients. L'oxygénothérapie était réalisée chez six patients (46%). La durée moyenne d'hospitalisation variait entre huit et trente jours avec une moyenne de quinze jours. Nous avons eu douze cas de guérison et un décès à la suite d'un sepsis sévère sur aplasie médullaire.

**Tableau 2:** Germes causales et leurs sensibilités à l'antibiogramme

Germes causales	Nombre de cas	Antibiotiques sensibles utilisés
Streptococcus agalactiae B	04	Amoxi-clavulanate
Streptococcus intermedius/milleri	09	Amoxi-clavulanate
Klebsiella pneumoniae		Ciprofloxacine
Staphylococcus auricularis méti R	03	Teicoplanine/ ciprofloxacine
Enterococcus faecalis	01	Amoxi-clavulanate
Escherichia coli	02	Ciprofloxacine
Proteus vulgaris	01	Ciprofloxacine
Actinomyces israëli	02	Amoxi-clavulanate

## Discussion

La cellulite cervico-faciale grave est exceptionnelle, mais redoutable pouvant compromettre le pronostic vital [2, 3]. Elle pose un réel problème de prise en charge, à la fois couteuse et difficile, dont l'identification des germes responsables constitue la clé du traitement. Selon la littérature, elle se voit surtout chez l'adulte avec une prédominance masculine [1, 4, 5]. Nos résultats vont dans ce sens, l’âge moyen était de 35 ans. La prise d'anti-inflammatoires, le retard et/ou la prise en charge initiale inadéquate favorisent l'extension et la transformation d'une cellulite circonscrite en une cellulite grave. En effet en plus des facteurs favorisants classiques (immunodépression dans toutes ses formes: diabète, insuffisance rénale..), les anti-inflammatoires stéroïdiens et surtout non stéroïdiens constituent un facteur essentiel de cette transformation [5, 6, 7]. Dans notre série 54% des patients immunocompétents avaient pris des AI au début de la symptomatologie. A notre sens les AI sont un facteur déterminant voir majeur dans la transformation d'une forme circonscrite en une cellulite grave. Une revue de la littérature montre que la prise des AI est retrouvée dans toutes les séries et leurs utilisations est déconseillée voire même dangereuse [1, 5, 6, 8]. Cela s'explique par le fait que la prise d'AI surtout non stéroïdiens (AINS), en absence d'antibiotique efficace, masque ou diminue la symptomatologie et favorise la diffusion de l'infection. Ils ont un effet dépressif sur les mécanismes humoraux de défense immunitaire contre l'infection [5, 9]. Ils réduisent de façon significative la synthèse des immunoglobulines G, inhibent l'adhérence des polynucléaires donc la phagocytose et la capacité phagocytaire des macrophages et augmentent la production des cytokines et leurs conséquences locales [10, 11]. La porte d'entrée est dentaire ou péri-dentaire dans plus de 80% des cas [11–13]. Cette incidence élevée est due à la mauvaise hygiène bucco-dentaire [1, 3, 11]. Le diagnostic est généralement clinique et doit permettre de distinguer deux formes de cellulites graves: la forme pseudo-phlegmoneuse ou phlegmon, du plancher buccal ou de la loge parotido-massétérine, qui se traduit par un processus inflammatoire douloureux, un trismus et des signes compressifs des voies aèro-digestives supérieures; et la forme gangréneuse avec une crépitation neigeuse sous cutanée, des nécroses et une extension rapide cervico-médiastinale [1, 2, 12]. Nous avons constaté que certains signes cliniques: trismus, dysphagie, douleur surtout thoracique, crépitations neigeuses et nécrose cutanée, constituent des signes de gravités de la cellulite. Sur le plan biologique, il y'a une hyperleucocytose modérée à prédominance de polynucléaires neutrophiles [1]. L'infection est polymicrobienne mixte, et si la prédominance des germes anaérobies fait l'unanimité des auteurs [2, 3, 9, 10, 11], la virulence microbienne est caractéristique de la cellulite grave avec au moins la présence d'un germe multi-résistant à l'antibiogramme.

Le traitement est médico-chirurgical associé à une réanimation adaptée [1, 9, 14]. Les investigations para cliniques ne doivent en aucun cas retarder la prise en charge. L'antibiothérapie doit être efficace et ciblée, elle est d'abord probabiliste visant le streptocoque et les anaérobies puis adaptée aux données de l'antibiogramme [6, 7, 15]. La durée est variable selon le degré de gravité et l’évolution. Le drainage et/ou le débridement chirurgical est un volet essentiel et doit permettre la mise à plat de toutes les zones cellulitiques par une voie d'abord large [1, 14–17]. Sans oublier l'identification et le traitement de la porte d'entrée [1, 4, 16]. En cas de médiastinite le drainage peut se faire par thoracotomie [3]. Le recours à la trachéotomie est justifié dans les collections rétro-pharyngées [18]. L'oxygénothérapie hyperbare est un complément du traitement chirurgical [14]. Elle est d'un apport capital grâce à son effet bactériostatique et son pouvoir de régénération tissulaire, cependant ses contre-indications et sa disponibilité limitent son utilisation [14]. Le pronostic de ces cellulites est lié essentiellement au terrain; la précocité et l'efficacité du traitement initial, dont l'isolement des germes en causes, constitue une étape déterminante. Le pourcentage de décès dans la littérature varie entre 7 et 50% [1, 19], dans notre série il était de 7%.

## Conclusion

Les cellulites cervico-faciales graves sont rares mais redoutables et posent un réel problème de prise charge thérapeutique. L'analyse des facteurs favorisants et l’évaluation des critères de gravité peuvent limiter une évolution défavorable.
